# *Sanguis draconis*, a Dragon's Blood Resin, Attenuates High Glucose-Induced Oxidative Stress and Endothelial Dysfunction in Human Umbilical Vein Endothelial Cells

**DOI:** 10.1155/2014/423259

**Published:** 2014-06-02

**Authors:** Yi Chang, Ting-Chen Chang, Jie-Jen Lee, Nen-Chung Chang, Yung-Kai Huang, Cheuk-Sing Choy, Thanasekaran Jayakumar

**Affiliations:** ^1^Department of Anesthesiology, Shin Kong Wu Ho-Su Memorial Hospital, 95 Wen-Chang Road, Taipei 101, Taiwan; ^2^School of Medicine, Fu-Jen Catholic University, 510 Zhong-Zheng Road, Taipei 205, Taiwan; ^3^Graduate Institute of Medical Sciences, College of Medicine, Taipei Medical University, Taipei 110, Taiwan; ^4^Department of Lymphatic Vascular Surgery, Wan Fang Hospital, Taipei Medical University, Taipei 110, Taiwan; ^5^Department of Pharmacology, Taipei Medical University, Taipei 110, Taiwan; ^6^Department of Surgery, Mackay Memorial Hospital, Taipei 104, Taiwan; ^7^Mackay Junior College of Medicine, Nursing, and Management, Taipei 112, Taiwan; ^8^Department of Cardiology, School of Medicine, Taipei Medical University, Taipei 110, Taiwan; ^9^School of Oral Hygiene, College of Oral Medicine, Taipei Medical University, Taipei 110, Taiwan; ^10^Emergency and Intensive Care Department, Min-Sheng Hospital 168, Taoyuan 330, Taiwan; ^11^Department of Medicine, Taipei Medical University, Taipei 110, Taiwan

## Abstract

Hyperglycaemia, a characteristic feature of diabetes mellitus, induces endothelial dysfunction and vascular complications by limiting the proliferative potential of these cells. Here we aimed to investigate the effect of an ethanolic extract of *Sanguis draconis* (SD), a kind of dragon's blood resin that is obtained from *Daemonorops draco* (Palmae), on human umbilical vein endothelial cells (HUVEC) under high-glucose (HG) stimulation and its underlying mechanism. Concentration-dependent (0–50 **μ**g/mL) assessment of cell viability showed that SD does not affect cell viability with a similar trend up to 48 h. Remarkably, SD (10–50 **μ**g/mL) significantly attenuated the high-glucose (25 and 50 mM) induced cell toxicity in a concentration-dependent manner. SD inhibited high glucose-induced nitrite (NO) and lipid peroxidation (MDA) production and reactive oxygen species (ROS) formation in HUVEC. Western blot analysis revealed that SD treatments abolished HG-induced phosphorylation of extracellular signal-regulated kinase 1/2 (ERK 1/2), nuclear transcription factor, **κ**B (NF-**κ**B), VCAM-1, and E-selectin, and it also blocked the breakdown of PARP-116 kDa protein in a dose-dependent manner. Furthermore, we found that SD increased the expression of Bcl-2 and decreased Bax protein expression in HG-stimulated HUVEC. Thus, these results of this study demonstrate for the first time that SD inhibits glucose induced oxidative stress and vascular inflammation in HUVEC by inhibiting the ERK/NF-**κ**B/PARP-1/Bax signaling cascade followed by suppressing the activation of VCAM-1 and E-selectin. These data suggest that SD may have a therapeutic potential in vascular inflammation due to the decreased levels of oxidative stress, apoptosis, and PARP-1 activation.

## 1. Introduction

Vascular disorders through overexpression of adhesion molecules are thought to play in the pathogenesis of atherosclerosis. Adhesion molecules are proteins which regulate the interaction between endothelium and leukocytes. An increase in their expression on the endothelial surface causes increased adhesion of leukocytes. Endothelial cells in human atherosclerotic lesions have been shown to overexpress intercellular adhesion molecule-1 (ICAM-1), vascular cell adhesion molecule-1 (VCAM-1), and E-selectin [1], and adhesion molecules are reported to activate by nuclear transcription factor-**κ**B (NF-**κ**B). Previous studies have shown that high glucose activates NF-**κ**B, one of the transcription factors for proinflammatory genes. NF-**κ**B is present in the cytoplasm as an inactive form bound to its inhibitor molecule, inhibitory factor of NF-**κ**B-*α* (I**κ**B-**α**). Translocation of NF-*κ*B from the cytoplasm to the nucleus is preceded by the phosphorylation, ubiquitination, and proteolytic degradation of I**κ**B-**α** [2]. Moreover, during the oxidative stress, endothelial cells generate ROS, such as superoxides and peroxynitrite, leading to low-density lipo protein (LDL) oxidation, and the formation of ROS together with inflammatory factors including chemokines, cytokines, and adhesion molecules has been shown to be increased in atherosclerotic lesions [3]. Hyperglycemia induces mitochondrial superoxide production and prevents activity and expression of endothelial nitric oxide synthase (eNOS) in endothelial cells [4]. ROS can modify endothelial function by a variety of mechanisms, such as peroxidation of membrane lipids, activation of NF-**κ**B, and decreasing the availability of nitric oxide (NO) [5].

Poly (ADP-ribose) polymerase (PARP), an abundant nuclear enzyme, initiates an energy consuming cellular metabolic cycle by transferring ADP-ribose units from NAD+ and ATP to nuclear proteins that leads to cellular metabolic disturbances and culminates in endothelial dysfunction [6]. It has been demonstrated that hyperglycemia induces PARP activation in endothelial cells in culture as well as in the vasculature of diabetic animals [6]. Recent studies have also established that activation of PARP plays a pivotal role in the overexpression of adhesion molecules and cytokines [7] and also on peroxynitrite formation [8]. In addition, a study has suggested that activation of PARP-1 is reported to be associated with hyperglycemia-induced ROS formation, as it is evidenced that PARP inhibitors blocked ROS production [9]. These observations lead to the suggestion that PARP-1, CAMs, ROS, and NF-**κ**B inhibitors could be used as a therapeutic strategy in diabetic complications.

*Sanguis draconis* (SD) is a kind of red resin that is obtained from several botanical origins, and most SD that is traded internationally is from* Daemonorops* [10]. SD has long been used as a traditional Chinese medicine for improving blood circulation, stopping hemorrhages, and healing wounds and cuts and is also used as an antiseptic [11]. Hou et al. have reported that SD can ameliorate the progress of insulin resistance and enhance insulin sensitivity [12], and it has been elucidated that SD could efficiently reduce diabetics by inhibiting high plasma lipid level [13] and intestinal carbohydrate absorption [14]. On the other hand, SD has also found to inhibit platelet aggregation, thrombus formation, and myocardial ischemia [15]. Our previous studies have described that SD inhibits streptozotocin-induced iNOS protein expression, pancreatic injury, and lipid peroxidation via the inhibition of NF-**κ**B activation [16], and it also inhibits the production of NO and prostaglandin E2 (PGE2) by downregulating iNOS and COX-2 gene expression via the suppression of NF-*κ*B (p65) activation [17]. We postulated that SD can retreat the effects induced by high glucose concentration in endothelial cells; therefore, we evaluated the effect of SD on ROS, NO, and MDA production and expression of adhesion molecules NF-*κ*B, PARP-1, ERK, and on Bax-Bcl2 in HUVEC treated with high concentrations of glucose.

## 2. Materials and Methods

### 2.1. Preparation of the Ethanolic Extract of* S. draconis*

Commercially available plant material (*Sanguis draconis*) was purchased from a traditional Chinese medicine drug store and the authenticity of SD was confirmed by Professor Ching-Chiung Wang of the School of Pharmacy, Taipei Medical University. A certificate of source and specimen is kept at our lab. Other details of preparation of SD are described in our previous paper [17].

### 2.2. Chemicals and Reagents

Cell culture reagents including M-199 medium, L-glutamine, penicillin, streptomycin, and fetal bovine serum were obtained from Gibco BRL (Grand Island, NY, USA). Anti-mouse and anti-rabbit immunoglobulin G-conjugated horseradish peroxidase (HRP) was purchased from Amersham Biosciences (Sunnyvale, CA, USA) and/or Jackson-ImmunoResearch (West Grove, PA, USA). Anti-eNOS, anti-p-NF-*κ*B, anti-cleaved PARP, anti-VCAM-1, anti-Bax, and Bcl2 were all purchased from Santa Cruz Biotechnology (Santa Cruz, CA, USA). The anti-p42/p44 ERK (Thr202/Tyr204) was from Cell Signaling (Beverly, MA, USA). The Hybond-P polyvinylidene difluoride (PVDF) membrane, enhanced chemiluminescence (ECL) Western blotting detection reagent, and analysis system were obtained from Amersham (Buckinghamshire, UK). All other chemicals used in this study were of reagent grade.

### 2.3. Isolation and Culture of HUVEC

Human umbilical cords were obtained from the Hospital of National Taiwan University, Taipei, Taiwan, and human umbilical vein endothelial cells were isolated by enzymatic digestion as described previously [18]. After 15-min incubation with 0.1% collagenase at 37 ± 0.5°C, umbilical cord vein segments were perfused with 30 mL of medium 199 containing 10 U/mL penicillin and 100 *μ*g/mL streptomycin for the collection of cells. After centrifugation for 8 min at 900 ×g, the cell pellet was resuspended in previous medium supplemented with 20% heat-inactivated fetal bovine serum, 30 *μ*g/mL endothelial cell growth supplement, and 90 *μ*g/mL heparin. Confluent primary cells were detached by trypsin, EDTA (0.05% : 0.02%, v/v), and passages between three and five were used in the experiments. Cultures had typical cobblestone morphology and stained uniformly for human von Willebrand factor (vWF) [19] as assessed by indirect immunofluorescence.

### 2.4. Cell Viability Assay

The viability of HUVECs upon treatment of glucose, SD alone, and combined together was measured by a colorimetric MTT assay. Briefly, HUVECs (2 × 10^5^ cells/well) were seeded on 24-well plates and cultured in DMEM containing 10% FBS for 24 h. HUVECs were treated with glucose at concentrations of (5.5–150 *μ*M) and SD (10–50 *μ*g/mL) alone and pretreated with SD (10–50 *μ*M) in glucose (25 and 50 mM) induced cells or an isovolumetric solvent control (0.1% DMSO) for 24 or 48 h. The cell number was measured based on the ability of mitochondria in viable cells to reduce MTT as previously described [17]. The cell number index was calculated as the absorbance of treated cells/control cells × 100%.

### 2.5. Measurement of Intracellular ROS

Starved HUVECs (2 × 10^5^ cells/well) were loaded with DCF-DA (20**μ**M) for 20 min. After treatment with SD (50 *μ*g/mL) for 2 hr, cells were stimulated with glucose (25–75 mM) for 24 hr, washed with PBS, and then detached using trypsin. Levels of intracellular ROS were detected by flow cytometry (Beckman Coulter). All experiments were repeated at least four times to ensure reproducibility.

### 2.6. Determination of Nitrite Production

HUVECs cultured in 12-well plates were washed twice with Hanks balanced salt solution (HBSS) and then incubated at 37 ± 0.5°C in the same buffer for 30 min with various concentrations of SD. Acetylcholine (30**μ**M) was used as a positive control. Supernatants were collected and then injected into a nitrogen purge chamber containing vanadium(III) chloride in hydrochloric acid at 91 ± 0.5°C. All NO metabolites can be liberated as gaseous NO and reacted with ozone to form activated nitrogen dioxide that is luminescent in red and infrared spectra. The chemiluminescence was detected using a nitric oxide analyzer (NOA280, Sievers Instruments, Boulder, CO, USA) [20]. For calibration, the area under the curve was converted to nanomolar NO using a NaNO_3_ standard curve, and the final data were expressed as* p*mol/mg protein.

### 2.7. Lipid Peroxidation Assay

Lipid peroxidation was assayed by the thiobarbituric acid (TBA) reaction method. The cells were homogenized in ice-cold 1.15% KCl. The samples were used to measure the malondialdehyde (MDA) formed in a peroxidizing lipid system. The amount of thiobarbituric acid reactive substance (TBARS) was determined using a standard curve of 1,1,3,3-tetramethoxypropane.

### 2.8. Western Blot Analysis

Western blot analysis was performed as previously described [21]. Lysates from each sample were mixed with 6× sample buffer (0.35 M Tris, 10% w/v SDS, 30% v/v glycerol, 0.6 M DTT, and 0.012% w/v bromophenol blue, pH 6.8) and heated to 95°C for 5 min. Proteins were separated by electrophoresis and transferred onto polyvinylidene difluoride (PVDF) membranes for pERK1/2, p-eNOS, pNF-*κ*B, VCAM-1, PARP, Bax, and Bcl2. The membranes were blocked with 5% nonfat milk in TBS-0.1% Tween 20 and sequentially incubated with primary antibodies and HRP-conjugated secondary antibodies followed by enhanced chemiluminescence (ECL) detection (Amersham Biosciences). BIO-PROFIL Bio-1D light analytical software (Vilber Lourmat, Marne La Vallee, France) was used for the quantitative densitometric analysis. Data of specific protein levels are presented as relative multiples in relation to the control.

### 2.9. Statistical Analyses

The experimental results are expressed as the mean ± SEM and are accompanied by the number of observations. For analysis of the results, a one-way analysis of variance (ANOVA) test was performed using Sigma Stat v3.5 software. When group comparisons showed a significant difference, the Student-Newman-Keuls test was used. A *P* value of <0.05 was considered to be statistically significant.

## 3. Results

### 3.1. Effects of SD on HUVEC Cell Viability

**Figure 1:**
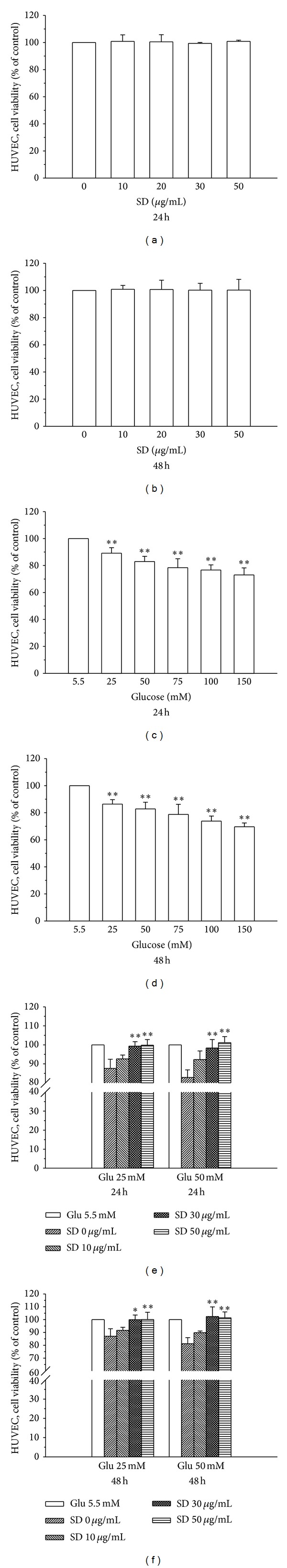
Effects of SD on the cell viability of human umbilical vein endothelial cells (HUVEC): ((a), (b)) the viability of HUVECs during treatment with various concentrations (10–50 *μ*g/mL) of SD for 24 and 48 h; ((c), (d)) the viability of HUVECs during treatment with various concentrations (25–150 mM) of glucose for 24 and 48 h; ((e), (f)) the viability of HUVECs upon treatment with various concentrations (0–50 *μ*g/mL) of SD in glucose-induced (25 and 50 mM) HUVECs for 24 and 48 h. Data are shown as the mean ± SEM of three independent experiments.*P < 0.05; **P < 0.01, compared with the glucose treated group.

**Figure 2:**
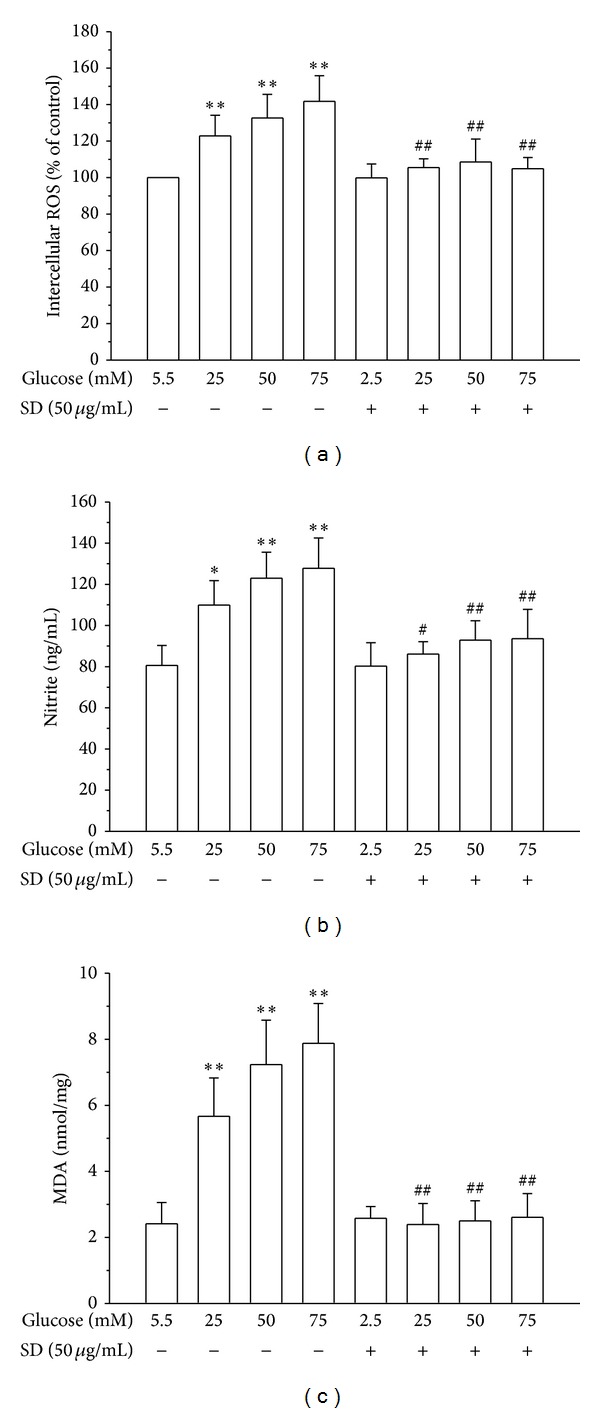
Effects of SD on HG-induced formation of ROS, NO, and LPO in HUVECs: (a) ROS production was determined as described in Materials and Methods; (b) the nitrite concentration in the culture medium was determined by Griess reagent; and (c) lipid peroxidation was assayed by measuring the amount of TBARS formation (malondialdehyde, MDA); Data are shown as the mean ± SEM of three independent experiments. *P < 0.05; **P < 0.01, compared with the normal group; ^#^ *P* < 0.05; ^##^ *P* < 0.01, compared with the HG-treated group.

The cytotoxic effect of various concentrations of SD and glucose on HUVECs cells was measured by MTT assay. The results indicated that SD did not affect the viability of HUVEC at the used concentrations of 0–50 *μ*g/mL for 24 and 48 h (Figures 1(a) and 2(b)). However, treatment of cells with glucose (5.5–150 *μ*M) decreased the cell viability of HUVEC in a concentration dependent manner. Interestingly, cells simultaneously incubated with glucose (25 and 50 mM) and SD (0–50 *μ*g/mL) increased cell viability in a concentration dependent manner (Figures 1(c), 1(d), 1(e), and 1(f)).

### 3.2. SD Inhibited HG-Induced ROS and NO Production

To investigate the effectiveness of SD in inhibiting HG-induced ROS formation in HUVECs, a cell-permeative ROS-sensitive dye, DCFDA (nonfluorescent in a reduced state but fluorescent upon oxidation by ROS), was used. The intracellular level of ROS in HUVECs increased concentration dependently following incubation with high glucose (25–75 mM) compared with 5.5 mM glucose. Incubation of HUVECs with SD resulted in a marked reduction of HG-induced intracellular ROS generation (Figure 2(a)). Contrary to our expectations, glucose increased NO production instead of reducing it; however, when HUVECs were treated with SD plus glucose, SD completely abrogated the production of NO induced by glucose (Figure 2(b)).

### 3.3. SD Inhibited HG-Induced MDA Production

Studies have indicated that damaging cell membranes may cause a decrease of cell viability through peroxidation of membrane lipids. Therefore, we projected to estimate the levels of MDA in the present study. As shown in Figure 2(c), MDA, a marker of lipid peroxidation, was markedly elevated in HG-induced HUVEC. However, treatment of SD (50 *μ*g/mL) significantly attenuated the elevation of MDA concentration in HG-stimulated HUVEC.

### 3.4. SD Enhanced the Phosphorylation of eNOS

**Figure 3:**
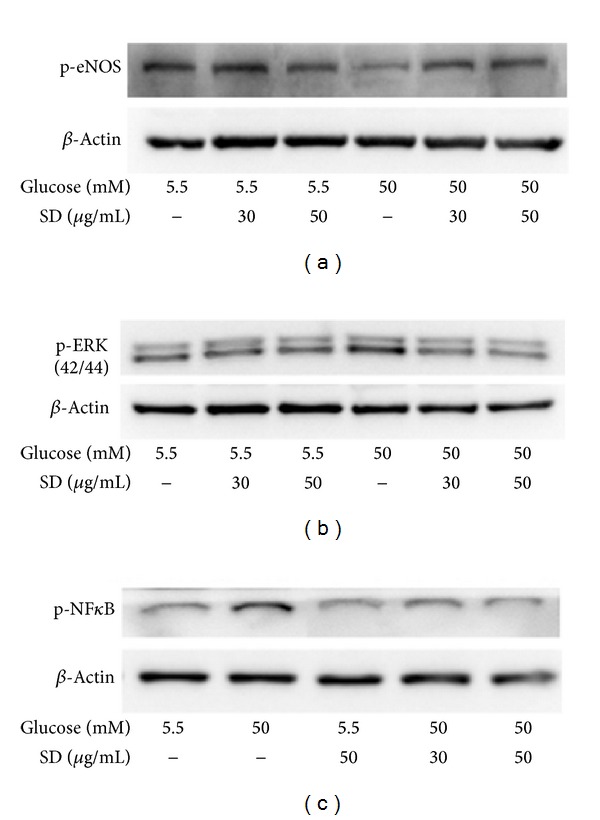
Effects of SD on HG-induced phosphorylation of eNOS, ERK, and NF-*κ*B in HUVECs: HUVECs (2 × 10^5^ cells/well) were pretreated with SD (30 and 50 *μ*g/mL) for 2 h and then treated with glucose (50 mM) for 30 min to detect the phosphorylation of (a) eNOS, (b) ERK1/2, and (c) NF-*κ*B. The *β*-actin was used as an internal control.

The result showed that SD (30 and 50 *μ*g/mL) alone had no influence on the protein expressions of phosphorylated eNOS at Ser1177, whereas high glucose leads to a significant decrease in the expression of eNOS (Figure 3(a)). Treatment with SD significantly attenuated the decreased level of eNOS expression.

### 3.5. SD Inhibited the Phosphorylation of ERK and NF-*κ*B

To determine whether SD affects the activation of the MAPK pathway, we analyzed the phosphorylation levels of pERK MAPK. First, HUVECs were pretreated with SD (30 and 50 *μ*g/mL) for 30 min and then stimulated with 50 mM glucose for 1 h. The HG-induced increased phosphorylation of pERK was inhibited by SD in a concentration-dependent manner (Figure 3(b)) and restored it to the level in cells exposed to 5.5 mM glucose. Moreover, we measured NF-*κ*B activation in HG-induced HUVECs, as it is suggested that increased ROS production in HG-induced HUVEC may partially cause the activation of NF-*κ*B. As we expected, the expression of NF-*κ*B was increased markedly in HUVEC cells treated with high glucose (50 mM). In addition, pretreatment with SD (30 and 50 *μ*g/mL) markedly inhibited the HG-induced expression of NF-*κ*B concentration dependently (Figure 3(c)).

### 3.6. Effects of SD on HG-Induced Endothelial Cell Adhesion Molecules

**Figure 4:**
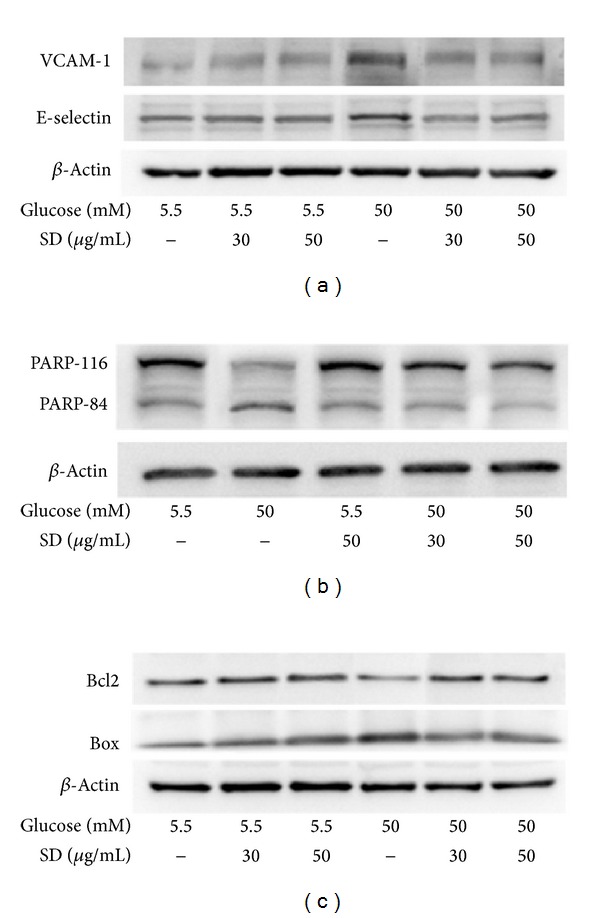
Effects of SD on HG-induced expression of VCAM-1, E-selectin, PARP-1, and Bax/Bcl2 in HUVEC: HUVECs (2 × 10^5^ cells/well) were pretreated with SD (30 and 50 *μ*g/mL) for 2 h and then treated with glucose (50 mM) for 30 min to detect the expression of (a) VCAM-1 and E-selectin, (b) PARP-1, and (c) Bax/Bcl2. The *β*-actin was used as an internal control.

To examine whether glucose induces expression of VCAM-1 and E-selectin in HUVECs, we cultured HUVECs at normal glucose (5.5 mM) and high glucose (50 mM) concentrations for 24 h. Immunoblot analysis showed that stimulation of HUVECs with high concentrations of glucose increased the production of VCAM-1 and E-selectin (Figure 4(a)). To further determine whether SD can inhibit the expression of endothelial adhesion molecules, HUVECs were pretreated with SD at concentrations of 30 and 50 *μ*g/mL and stimulated with 50 mM glucose for 24 h. As shown in Figure 4(a), pretreatment of SD in HUVEC significantly inhibited the HG-induced expression of ICAM-1 and E-selectin in a concentration manner.

### 3.7. Effects of SD on HG-Induced PARP-1 Overactivation

As previously described, the full PARP-1 protein is 116 kDa, and the 85 kDa cleavage product is an early marker of apoptosis. In this experiment, we detected both the 116 kDa full PARP protein and the 85 kDa breakdown product. The results revealed that exposure of HUVEC to high glucose concentrations (50 mM) for 24 h resulted in a significant decrease in the amount of PARP-116 kDa protein and increased their breakdown product of 85 kDa protein. Interestingly, treatment of SD alone significantly enhanced the amount of PARP-116 full protein in HUVEC. Moreover, the amount of 85 kDa PARP breakdown product induced by high glucose (50 mM) was markedly reduced in SD treated cells in a concentration dependent manner (30 and 50 *μ*g/mL) (Figure 4(b)).

### 3.8. Effects of SD on HG-Induced Bcl-2/Bax Expression

As shown in Figure 2(a), the inhibition of HG-induced ROS by SD led us to evaluate any possible effects of SD on other mediators of oxidative stress-induced apoptosis. The results revealed that the ratio between the anti- and proapoptotic mediators Bcl2 and Bax was not much affected by SD treatment alone, especially that Bcl-2 did not alter between normal and SD treated cells (Figure 4(c)). Moreover, HG-induced Bax was moderately reversed by SD, which indicated that Bax might play a role in this effect.

## 4. Discussion

In diabetes, hyperglycemia causes vascular complications that are produced mainly by the overproduction of ROS [22]. Dysfunction and activation of the endothelium are regarded as important factors in the pathogenesis of vascular disease in diabetes mellitus [23]. In this work, we evaluated the protective effect of* Sanguis draconis* (SD), a kind of red resin, against the dysfunction and activation of endothelial cells induced by high concentrations of glucose. The major finding of this study is that SD was capable of attenuating the increase of ROS, NO, and MDA and inhibits overactivation of PARPs, VCAM-1, NF-*κ*B, and Bcl2 in cells exposed to high glucose concentration, and our data contribute in part to elucidate the molecular mechanisms involved in this effect.

Oxidative stress, a hallmark of high glucose-induced endothelial dysfunction, was significantly attenuated by SD treatment in the current study. There is growing evidence that oxidative stress is involved in the pathogenesis of diabetic complications [24] and an acute increase of glycemia is reported to be accompanied by oxidative stress generation [25]. Lipid peroxidation (LPO), a process induced by free radicals, leads to oxidative deterioration of polyunsaturated lipids. Malondialdehyde (MDA), a secondary product of lipid peroxidation, is used as an indicator of tissue or cell damage. In previous studies, it was found that MDA level was increased in HUVECs when cells were incubated with glycated protein and iron [26]. An increased production of ROS has been reported in inflammatory responses, and it acts as a potential mediator of diabetes mellitus-associated vascular diseases. ROS is also considered to be of the important mediators of several biologic responses, including cell proliferation and extracellular matrix deposition. A recent study showed that hyperglycemia stimulates the generation of free radicals and oxidative stress in various cell types [27]. In this study, an elevation in HG-mediated production of cellular MDA and ROS indicate that oxidative stress induced by high glucose in HUVEC is important in determining the character of diabetic complication as well as vascular inflammation. Interestingly, we found that SD treatment potentially inhibited the high glucose-induced MDA and ROS formation, suggesting a role of protecting oxidative insults in HG-induced HUVEC.

Bioavailability of endothelial NO observed in individuals with hyperglycemia is considered to be a critical and initiating factor in the pathogenesis of diabetic vascular complications [28]. NO is synthesized in endothelial cells from the substrate L-arginine via endothelial NO synthase (eNOS) [26], and this enzyme plays an important role for the maintenance of cardiovascular function by producing NO synthesis. Nevertheless, this enzyme can be detached, leading to the generation of superoxide instead of NO under certain pathological circumstances and oxidative stress conditions [29]. Our previous study showed that SD inhibits NO production induced by IL-1**β**/IFN-**γ** in endothelial cells [17]. The results of this study revealed that high glucose causes a significant degree of oxidative stress, which leads to increases in the formation of NO; convincingly pretreatment of SD could reverse the effect of high glucose by suppressing NO production. These results indicate that SD may have a protective effect on HUVEC by preventing or decreasing the injury of endothelial cells by interfering with NO synthase and NO.

In HUVEC, high glucose-induced cellular damage could lead to cell apoptosis probably viaERK activation. To make this hypothesis solid, we next aimed to gain insight into the cell signaling pathways mediating the action of SD on HG-induced human endothelial cells. We found that HG potentiated the activation of both ERK1/2 and NF-*κ*B, indicating that overactivation of endothelial signaling molecules can be triggered by HG stimulations. ERK is the signal cascade involved in the protection of oxidative damage and its activation is generally thought to mediate cell survival [30]. In agreement with our study, a previous study has shown that high D-glucose activates the ERK1/2, NF-*κ*B, and* i*NOS when this signaling pathway is formerly triggered by an exogenous inflammatory stimulus in cultured HUVEC [31]. Chronic activation of NF-*κ*B is associated with various pathological conditions, including insulin resistance. Human subjects with type 2 diabetes exhibit increased activity of NF-*κ*B in muscle that directly correlates with impaired insulin mediated glucose disposal. Enduring hyperglycemia and/or extreme perturbations in glycemia are common generators of oxidative stress, which has been shown to induce insulin resistance through activity of NF-*κ*B. Interestingly, administration of the common salicylate aspirin (7.0 g/day) in patients with type 2 diabetes enhances glucose homeostasis and peripheral insulin sensitivity, at least in part through the inhibition of NF-*κ*B nuclear activity [32]. Targeted manipulation or ablation of NF-*κ*B, therefore, remains at the forefront of innovative treatments for diabetes and inflammatory pathologies. Our study also shows that high glucose elevated the expression of NF-*κ*B in HUVEC and these effects were blocked by SD treatment. Changes in expression of activated ERK and NF-*κ*B closely reflect the cell damage; such results suggest that high glucose-induced HUVEC cell damage is protected, at least partly, by inhibiting ERK and NF-*κ*B activation.

In agreement with others [33], we have further found that ERK1/2 and NF-*κ*B activation was responsible for endothelial VCAM-1 and E-selectin induction. Other signaling molecule, like PARP-1, has been reported as promoters of ICAM-1 expression in different vascular cell types [34] and might therefore be involved in the induction of ICAM-1 in HG-induced HUVEC. Adhesion induced by glucose was almost completely abrogated by SD when added before the addition of glucose, indicating that SD can have a preventive and protective effect against the endothelial activation induced by hyperglycemia. SD treatment was also capable of decreasing the glucose-induced expression of VCAM-1 and E-selectin. Similar results have been described in a previous study in endothelial cells, where they have reported that dehydroepiandrosterone (DHEA), an adrenal steroid abrogated expression of adhesion molecules in HUVEC, activated with either TNF-**α** or oxidized low density proteins [35]. Recently, various phytochemicals have been shown to inhibit the expression of adhesion molecules in endothelial cells. For instance, epigallocatechin-3-O-gallate (EGCG) inhibits angiotensin II-induced adhesion molecule expression by inhibiting p38 MAPK and ERK1/2 phosphorylation [36]. Anthocyanins inhibited TNF-**α**-induced ICAM-1 and VCAM-1 expressions via the NF-*κ*B-dependent pathway [37]. Phloretin inhibited the TNF-**α**-stimulated expression of adhesion molecules without activating NF-*κ*B [38]. Grape seed proanthocyanidin extract inhibited VCAM-1 expression in HUVECs via the NF-*κ*B-independent pathway [39]. In this study, the observed effect of SD on suppressing HG-induced expression of VCAM-1 and E-selectin may perhaps via inhibition of the ERK1/2 phosphorylation and NF-*κ*B-dependent pathway. Thus, development of therapeutic drugs for diabetic complications targeting CAMs expression may prove useful in the prevention of vascular inflammation.

Moreover, recent studies have demonstrated that activation of PARP is associated with the pathogenesis of diabetes and diabetic complications, including cardiovascular dysfunction. PARP has been shown to act as a coactivator in NF-*κ*B-mediated transcription [40]. Some studies have indicated that PARP inhibitor prevents the diabetes-induced elevation in circulating nitrite levels in streptozotocin-induced diabetes. A study has reported that PARP deficiency suppresses NF-*κ*B activation in cultured endothelial cells under high glucose stimulation [6], another study has also shown that NF-*κ*B is regulated by PARP in diabetic retinopathy [41]. In this study, it was found that high glucose induced breakdown of the full PARP protein-116 kDa in HUVECs. It has been demonstrated that PARP-1 inhibition attenuates the development of albuminuria and podocyte apoptosis and depletion in diabetic mice [42]. Our findings revealed that SD significantly abrogated HG-stimulated breakdown of the full protein PARP-116 in HUVEC. Furthermore, the ROS reducing effect of SD prompted us to verify whether this drug could reduce apoptosis rate by affecting other regulators of the oxidative stress-induced apoptosis, such as the anti- and proapoptotic Bcl2 family members Bcl2/Bax (Figure 4(c)). We noticed a significant variation in Bcl2/Bax ratio in HG compared to normal glucose-treated cells. However, SD treatment only affected Bax, as it significantly reduced their expression in HG-induced HUVEC cells. This result indicates that SD reduces HG-induced endothelial apoptotic rate via Bax inhibition, favoring the maintenance of the integrity of the endothelial cells and thus possibly contributing to reduce vascular inflammation. A previous study has also demonstrated that the ratio between Bcl2/Bax was not affected by fenofibrate, a PPAR*α* agonist, and also did not differ between normal and high glucose conditions [43].

In conclusion, the present work provides experimental evidence that* Sanguis draconis* (SD) could suppress high glucose-induced endothelial dysfunction and oxidative stress via inhibition of ERK/NF-*κ*B/PARP-1 activation in primary cultured HUVEC. Moreover, SD was found to exert an inhibitory effect against the HG-induced production of oxidative stress markers, including ROS, NO, and LPO, and the activation of VCAM-1 and E-selectin in HUVECs. Taken together, the results of this study may suggest that SD might be very useful in the treatment of diabetes mellitus vascular complications.

## Abbreviations

HG: High glucose
SD: Sanguis draconis
HUVEC: Human umbilical vein endothelial cells
NO: Nitrite
LPO: Lipid peroxidation
MDA: Malon-di-aldehyde
ROS: Reactive oxygen species
PARP: Poly-ADP ribose polymerase
NF-*κ*B: Nuclear transcription factor-*κ*B
VCAM-1: Vascular cell adhesion molecule-1.
